# Editorial: Gamete quality and assisted reproductive technology (ART) outcomes

**DOI:** 10.3389/fcell.2023.1152086

**Published:** 2023-02-22

**Authors:** Albert Salas-Huetos, Sergi Bonet, Jordi Ribas-Maynou

**Affiliations:** ^1^ Unit of Cell Biology, Department of Biology, Faculty of Sciences, University of Girona, Girona, Spain; ^2^ Biotechnology of Animal and Human Reproduction (TechnoSperm), Institute of Food and Agricultural Technology, University of Girona, Girona, Spain

**Keywords:** gamete, assisted reproductive technologies (ART), miRNA, DNA fragmentation, microbiota, fertility

One of the most astonishing processes in nature is the creation of a new individual from two single superspecialized cells; the oocyte and the sperm. Gamete quality and fertilization process is of great interest to the general population and has wide implications for human health. Although outstanding research has been in this area, many questions remain unanswered. For example, why reproductive health seems to be declining over the last few years? (GBD 2017 Population and Fertility Collaborators et al., 2018; Levine et al., 2017; Skakkebaek et al., 2019; Levine et al., 2022). This problem has resulted into an increase of around 20% in the total number of assisted reproductive technology (ART) cycles in the last two decades (de Mouzon et al., 2020). The current Research Topic on “*Gamete quality and assisted reproductive technology (ART) outcomes*” has collected cutting-edge original research on these key topics from an interdisciplinary perspective, representing the current research progress in this dynamic and central discipline. The Research Topic currently includes four papers.

Firstly, in “*MicroRNA-targeting in male infertility: Sperm microRNA-19a/b-3p and its spermatogenesis related transcripts content in men with oligoasthenozoospermia*,” Abu-Halima and collaborators add some spark to the role of sperm microRNAs (miRNAs) in infertility (Abu-Halima et al.). Several papers reported on the essential role of sperm miRNAs for spermatogenesis and early embryo development (Salas-Huetos et al., 2019; Salas-Huetos et al., 2020), however, the miRNA-mRNA interaction is not well understood yet for the vast majority of miRNAs. In the paper published on this Research Topic, the expression levels of both hsa-miR-19a-3p and -19b-3p were measured in 82 age-matched men (41 normozoospermic and 41 oligoasthenozoospermic). An upregulation of miR-19a/b-3p in oligoasthenozoospermic men was found. In an *in silico* prediction of miR-19a/b-3p target genes, 130 mRNA were identified and 82 were selected for RT-qPCR validation based on their role in sperm function or spermatogenesis. The authors described 51 target genes significantly downregulated in oligoasthenozoospermic men and, interestingly, suggest that the higher expression of both miRNAs (hsa-miR-19a-3p and -19b-3p) or the lower expression of target genes are associated with oligoasthenozoospermia and probably with male infertility.

Second, in a brief animal report, titled “*The capacity to repair sperm DNA damage in zygotes is enhanced by inhibiting WIP1 activity*,” the authors suggested a central role of wild-type p53-induced phosphatase 1 (WIP1) in paternal DNA reparation in zygotes (Leem et al.). In their research, Leem et al., added a specific WIP1 inhibitor (GSK2830371) in oocyte culture medium after the ICSI procedure and compared it with oocytes treated with DMSO (controls). While it was known that sperm DNA damage may be repaired after fertilization in zygotes, using maternal DNA repair factors (Fernández-Díez et al., 2016), here authors present the first evidence suggesting that WIP1 inhibition during fertilization reduces DNA damage in the paternal pronucleus. Therefore, it seems reasonable that supplementation of the culture medium with a WIP1 inhibitor would help correcting damaged DNA during ART procedures. Randomized clinical trials adequately designed to find the positive/negative consequences of this culture media supplementation in main ART outcomes are required to suggest a change in standardized ART practices.

Thirdly, in the paper entitled “*Seminal microbiota of idiopathic infertile patients and its relationship with sperm DNA integrity*,” García-Segura et al. investigated one of the newest associations described in the field: seminal microbiota and fertility. The aforementioned study not only characterized the seminal microbiota by sequencing the 16S rRNA in a well-characterized western Mediterranean population, but also evaluated its relationship to sperm chromatin integrity parameters (using TUNEL, Comet, and Chromomycin A3), and oxidative stress (using the MiOXSYS system). Researchers described *Firmicutes*, *Proteobacteria*, *Actinobacteria*, and *Bacteroidetes* as the most abundant phyla in seminal plasma and reported that their results were consistent to previous studies (Okwelogu et al., 2021; Yao et al., 2022). Moreover, looking for specific genera, the abundances of *Moraxella*, *Brevundimonas*, and *Flavobacterium* negatively correlate with sperm DNA fragmentation, *Brevundimonas* correlate with lower oxidative-reduction potential, and *Actinomycetaceae*, *Ralstonia*, and *Paenibacillus* correlated with a chromatin protamination status reduction and increased double-stranded DNA fragmentation. These novel findings support the hypothesis that the seminal microbiome may have an important role in male fertility, but further studies are needed to elucidate whether its effects can be overcomed.

Finally, Xu et al. investigated the “*Impact of elevated progesterone in late follicular phase on early pregnancy outcomes and live birth rate after fresh embryo transfers*” retrospectively in a large cohort of patients who underwent IVF/ICSI treatment cycles. It is well accepted that progesterone treatment is a good practice in ART cycles (Labarta and Rodríguez, 2020), but what about their concentration and timing? In summary, an increase in the serum progesterone level in the late follicular phase undermines main ART outcomes (e.g., pregnancy and live birth). Here, authors informed that the level of progesterone (1.5 ng/mL) is important for ART outcomes, but also evidenced that the timing may be essential to improve the effectiveness of the procedure. Further studies in multicentric and well-weighted cohorts are needed to definitely conclude progesterone timing and concertation in IVF cycles.

The wide range of subjects of this Research Topic in *Frontiers in Cell and Developmental Biology* shows the great effort of the scientific community in drawing a broad picture of the role of both male and female gametes in fertility and ART outcomes. In general, this Research Topic has revealed that sperm miRNAs, sperm DNA fragmentation, seminal microbiota, and time and concentration of progesterone during IVF/ICSI treatments are determinant factors for fertility. Incredible challenges and opportunities have been opened thanks to these contributions. To ensure continued progress in the field well-designed and well-funded studies are required to make significant strides.
